# Phytoremediation potential depends on the degree of soil pollution: a case study in an urban brownfield

**DOI:** 10.1007/s11356-023-26968-5

**Published:** 2023-04-28

**Authors:** Alicia Fernández-Braña, Lorena Salgado, José Luis R. Gallego, Elías Afif, Carlos Boente, Rubén Forján

**Affiliations:** 1 INDUROT and Environmental Biogeochemistry and Raw Materials Group, Campus de Mieres, Universidad de Oviedo, Mieres, Asturias Spain; 2 Department of Organisms and Systems Biology, Universidad de Oviedo, Mieres, Asturias Spain; 3 SMartForest Group, Department of Organisms and Systems Biology, Polytechnic School of Mieres, Universidad de Oviedo, Mieres, Asturias Spain; 4 Center for Research in Sustainable Chemistry (CIQSO), University of Huelva, Huelva, Spain

**Keywords:** Metal(loid), Phytostabilization, Phytoextraction, *A. pseudoplatanus*, *B. davidii*, *B. celtiberica*

## Abstract

Phytoremediation is a cost-effective nature-based solution for brownfield reclamation. The choice of phytoextraction or phytostabilization strategies is highly relevant when planning full-scale treatments. A suitable approach to identify such species involves the evaluation of plants that grow spontaneously on the contaminated sites. Here, we sought to determine the phytoremediation potential of three spontaneous plant species, namely the trees *Acer pseudoplatanus* L (*A. pseudoplatanus*) and *Betula celtiberica* Rothm. & Vasc (*B. celtiberica*), and the shrub *Buddleja davidii* Franch (*B. davidii*), for the recovery of an urban brownfield. To determine the response of the species to the degree of contamination, we conducted soil and vegetation sampling inside and outside the site. The concentrations of As, Cu, and Zn in soil and plant samples were measured, and then various indexes related to phytoremediation were calculated. The translocation factor and transfer coefficient indicated that vegetation outside the brownfield had phytoextraction capacity while the same plants inside the brownfield revealed phytostabilization properties. Given our results, we propose that the selected species are suitable for phytostabilization strategies in areas with high concentrations of contaminants, whereas they could be used for phytoextraction only in soils with low or moderate levels of pollution.

**Graphical Abstract:**

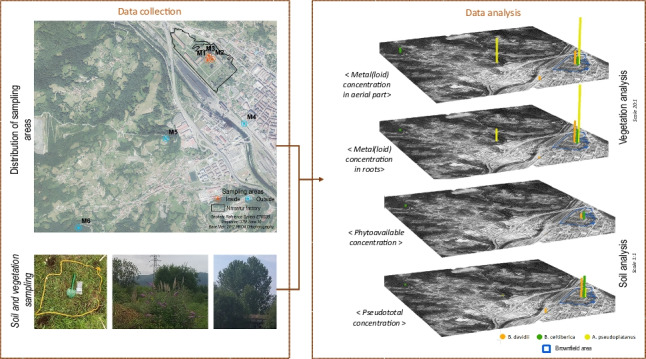

**Supplementary information:**

The online version contains supplementary material available at 10.1007/s11356-023-26968-5.

## Introduction

Urban brownfields are abandoned industrial sites close to inhabited areas. These brownfields may contain pollutants, thus significantly restricting land-use planning (O’Connor et al. 2019). However, they also offer strategic opportunities for the sustainable transition of metropolitan territories (Rey et al. 2022) as their remediation is essential to create new green zones. Reclamation of brownfield sites eliminates environmental risks and helps to reduce greenhouse gas emissions (Hou et al. 2018). In this context, phytoremediation has proved to be a cost-effective and environmentally friendly alternative to conventional soil remediation methods and it is included in the new trend of nature-based solutions (NBS) for environmental remediation (Guidi Nissim and Labrecque 2021). The use of phytoremediation for brownfield remediation enhances soil health helps to regulate urban temperature, improves urban hydrology, supports greater biodiversity, and attenuates air and noise pollution (Guidi Nissim and Labrecque 2021). The two most common phytoremediation options are phytostabilization and phytoremediation.

Plant species vary in their capacities to accumulate or tolerate metal(loid)s in aerial structures and roots, and this capacity is determined by the concentration of metal(loid)s present in the soil, by the physiological features of the species and by their selectivity for specific metal(loid)s (Massenet et al. 2021; Pilon-Smits 2005). Phytostabilization is a type of phytoremediation aimed at immobilizing pollutants in a contaminated substrate, by establishing vegetation on top of the polluted material (Forján et al. 2018). On the other hand, phytoextraction is a phenomenon in which hyperaccumulator plants absorb metals from the soil through the root system and translocate them to the harvestable shoot, making it possible to recover metals from the harvestable parts of plants (Forján et al 2017; Rodríguez-Vila et al. 2016).

A key aspect when implementing phytoremediation is the selection of the appropriate species. A common strategy is to use a plant that grows spontaneously and abundantly in the contaminated soil (Ali et al. 2013). Authors such as Mukhopadhyay et al. (2017) and Midhat et al. (2016) have shown that species that grow spontaneously in contaminated soils exhibited good phytoremediation behavior. Subsequently, the phytoremediation capacity, i.e., phytoextraction or phytostabilization properties of the potential candidates, should be evaluated (Forján et al. 2018). Indeed, several studies have shown that species and ecotypes present in metal(loid)-polluted sites tolerate high concentrations of soil pollutants and often show tolerance mechanisms that allow them to grow under these stress conditions (Schat et al. 2020).

Langreo (Spain) is an example of an area severely affected by heavy industry and mining activities. One of the most important activities of this area for decades was the production of fertilizers, which lead to the development of a 20 ha urban brownfield site named Nitrastur (Gallego et al. 2016; Gil-Díaz et al. 2016). Previous studies on this site revealed the presence of native herbaceous plants useful for phytostabilization purposes (Matanzas et al. 2021), whereas Mesa et al. (2017) focused on a specific study of enhanced phytoextraction via bioaugmentation; however, in those works, the main criteria that could be followed to design a real-scale phytoremediation were not addressed.

Following the previous considerations, the aim of this work was the study of the phytoremediation capacities and strategies followed by *Buddleja davidii* Franch (*B. davidii*), *Betula celtiberica* Rothm. & Vasc (*B. celtiberica*), and *Acer pseudoplatanus* L (*A. pseudoplatanus*), all of them growing abundantly in the study site and in the neighboring area. Of note, we addressed the different behaviors of these plants at different levels of soil pollution (very high inside the polluted site and much lower in the surroundings). Results will be helpful in the species selection for real-scale treatments, depending on the degree of soil pollution and the phytoremediation strategy to be followed (phytoextraction or phytostabilization).

## Material and methods

### Study area

**Fig. 1:**
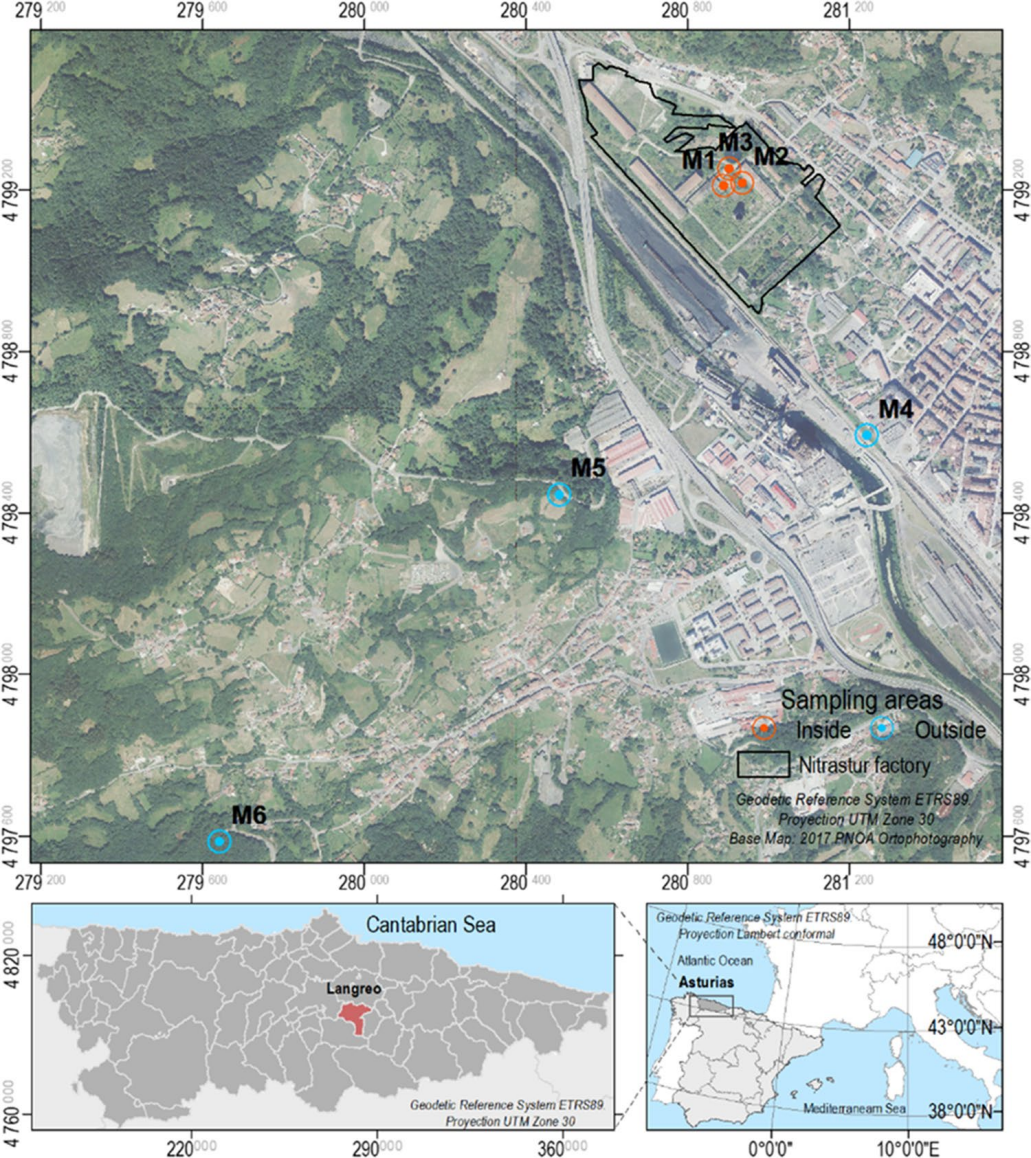
Study sites and sampling areas

The study area includes the urban brownfield of Nitrastur (20 ha)—which is colonized by a range of pollution-tolerant plants—and its surroundings, where some of the same species are also abundant (Fig. 1). Nitrastur was one of the main fertilizer plants in Spain; it is located in Langreo (Asturias) that has been an important industrial area since the nineteenth century, hosting activities such as coal mining and a coal-fired power plant, steel, and chemical industries. Most of these industrial and mining activities were abandoned in the last three decades leaving behind large amounts of waste that were disposed of in natural soil (see Gallego et al. 2016 and references therein).

Nitrastur is currently one of the largest brownfields in Spain and was included in the national inventory of polluted areas in 2001 and recently again in 2018. A detailed study (Gallego et al. 2016) revealed pyrite ashes, resulting from the roasting of pyrites for sulfuric acid production, as the main source of pollution whereas an assessment of site-specific human health risks (Wcislo et al. 2016) demonstrated the need for remediation, and thus, several attempts have been made (Baragaño et al. 2021). Within the brownfield, the values of pseudototal concentrations of As, Cu, Zn, and other elements usually exceed the limits established by the Spanish regulation in force (BOPA 2014), peaking up nowadays to thousands of mg·kg^−1^ in some areas.

### Soil and plant sampling design

Three of the predominant species were *Acer pseudoplatanus* L (*A. pseudoplatanus*), *Betula celtiberica* Rothm. & Vasc (*B. celtiberica*), and *Buddleja davidii* Franch (*B. davidii)*. Clusters of these plants were found in surrounding areas of the brownfield. The sampling was based on the simultaneous sampling of vegetation and soil (Fig. 1). The sampling stations were selected in locations in which several individuals of one of the target plants were found within a few square meters. The sampling locations were labelled M1, M2, and M3 (inside Nitrastur), and M4, M5, and M6 (outside). To build a composite sample for vegetation, six samples were taken from the aerial part and roots of individual plants belonging to the same species. For soil sampling, each sample consisted of four increments (1 kg) of the first 20 cm of soil, which was collected using a Dutch Edelman probe at each sampling point. This soil corresponded to the rhizosphere of the sampled vegetation. Soil samples were preserved in sterilized plastic bags and stored at 4 °C until preparation and analysis.

### Soil analysis

Soil pH was determined using a Mettler Toledo SevenCompact multimeter (1:2.5 water/soil). The organic matter content (OM) was determined by ignition (24 h–540 °C). Pseudototal metal(loid) concentrations were extracted with aqua regia (HCl + HNO_3_) in an Anton Paar 3000 microwave and measured by ICP-MS (Inductive Coupled Plasma Mass Spectrometer; ICP-MS 7700, Agilent Technologies). Phytoavailable concentrations of metal(loids)s were extracted by two methods to obtain more reliable data (Asensio et al. 2018; Lebourg et al. 2010; Menzies et al. 2007). In this regard, we performed one extraction with 0.01 M CaCl_2_ (Houba et al. 2008) and another with 0.1 M (NH_4_)_2_SO_4_ (Fresno et al. 2016). Metal(loid) concentrations were determined using the same ICP-MS device described above, and Standard Reference Material 1515 Apple leaves from NIST (National Institute of Standards and Technology) were used.

### Plant analysis and accumulation of metal(loid)s in plant tissues

Biomass was washed with deionized water, and fresh biomass was weighed. Dry biomass was assessed after oven-drying for 48 h at 80 °C and cooling at room temperature. Metal(loid) concentrations were quantified by Inductively ICP-MS (7700; Agilent Technologies, USA) after acid digestion (H_2_O_2_ and HNO_3_ (1:2 v/v)) in a microwave oven (Milestone ETHOS 1, Italy). The behavior of the metal(loid)s in the soil/plant system was addressed by examining the following parameters (Table 1):

- The translocation factor (TF), where a high value indicates a relatively high shoot metal concentration compared to its root concentration (Forján et al. 2018).
- The transfer coefficient (TC) in the studied plants measured their efficiency to take up metals from the soil (Rodríguez-Vila et al. 2014).
- The bioconcentration factor (BF) describes the ratio of available metal(loid) concentration that is taken up into shoots or roots. High BF values indicate a high concentration of elements in shoots or roots compared to the available concentration of the metal(loid)s (Rodríguez-Vila et al. 2015).

**Table 1:** Relation of soil/vegetation factors calculated

Factor	Expression	Classification	Reference
Translocation factor (TF)	$\frac{{C}_{a}}{{C}_{r}}$	TF > 1; plant translocation of metal(loid)s TF < 1; no plant translocation of metal(loid)s	Baker and Brooks 1989
Transfer coefficient (TC)	$\frac{{C}_{a}}{{C}_{s}}$	TC > 1; accumulator biosystem of metal(loid)s TC < 1; no accumulator biosystem of metal(loid)s	Busuioc et al. 2011; Peijnenburg and Jager 2003
Bioconcentration factor (BF)	$\frac{{C}_{a}}{{C}_{ex}}$	––	McGrath and Zhao 2003; Rodríguez-Vila et al. 2015
	$\frac{{C}_{r}}{{C}_{ex}}$		Rodríguez-Vila et al. 2015

*C*_*a*_, concentration of meta(loid)s in aerial part (mg kg^−1^); *C*_*r*_, concentration of meta(loid)s in roots (mg kg^−1^); *C*_*s*_, pseudo-total concentration of meta(loid)s in soil (mg kg^−1^); *C*_*ex*_, concentration of meta(loid)s extracted with (NH_4_)_2_SO_4_ (mg kg^−1^)

A high TF value indicates a relatively high shoot metal(loid) concentration compared to its root concentration; i.e., a plant species moves metal(loid)s effectively from the roots to shoots when the TF > 1. In contrast, TF values below 1 may indicate that the plant accumulates the contaminants in the root and thus acts as a phytostabilizer (Forján et al. 2018). In this regard, the ideal plant species for phytostabilization purposes are the “metal excluders,” which show a very low root-to-shoot TC (Kidd et al. 2009). This coefficient indicates efficiency to take up metals from the soil, and a plant is considered to be an accumulator biosystem whenever TC is higher than 1 (Busuioc et al. 2011). Finally, as regards BF, this parameter relates the extractable metal(loid) concentration in the soil to the concentrations in the aerial and root parts of the plant; BF is highly dependent on the method used to measure the extractable metal(loid) concentration (Karami et al. 2011), and thus, we have applied two different extractants ((NH_4_)_2_SO_4_ and CaCl_2_) to obtain the extractable metal(loid) concentration.

### Statistical analysis

The analytical determinations were performed in triplicate. Analysis of variance (ANOVA) and test of homogeneity of variance were carried out. In the case of homogeneity, a post hoc least significant difference (LSD) test was performed. If there was no homogeneity, Dunnett’s T3 test was performed. The Student’s *t*-test was used to compare the results of two samples at a time. A correlated bivariate analysis was also carried out using Pearson’s correlation. All data were processed with the statistical program SPSS (V.19).

## Results and discussion

### General characteristics of soils

The difference in pH between the samples taken inside and outside the urban brownfield was not relevant (Table 2). The soil with the lowest pH was M1, (pH 6.08), followed by M2. In this context, acidic pH values inside Nitrastur may have been caused by the presence of pyrite ash residues mixed with soil (Gallego et al. 2016). The rest of the pH values coincided with previous reports of slightly alkaline values (Baragaño et al. 2021). Regarding vegetation, the lowest pH values were observed in the soils hosting *B. davidii*, followed by *B. celtiberica* and *A*. *pseudoplatanus* (Table 2). As regards organic content, the soils inside the urban brownfield (M1, M2, M3) had a similar OM content, which was generally lower than that recorded outside the site. The soils M4 and M6 had the highest OM content, possibly because they had well-defined O and A horizons. In turn, these points were associated with *B. davidii* and *B. celtiberica* (Table 2).

**Table 2:** General soil characteristics: inside and outside the urban brownfield

Zone	Sample area	Plant species	pH	OM (%)
Inside	M1	*B. davidii*	6.08 ± 0.36b	5.17 ± 1.28c
	M2	*B. celtiberica*	6.82 ± 0.99ab	5.17 ± 1.84c
	M3	*A. pseudoplatanus*	7.41 ± 0.95a	5.33 ± 0.23c
Outside	M4	*B. davidii*	7.17 ± 0.08a	32.71 ± 0.88a
	M5	*A. pseudoplatanus*	7.92 ± 0.62a	3.91 ± 0.23c
	M6	*B. celtiberica*	7.79 ± 0.25a	17.31 ± 0.75b

Different letters for different samples indicate significant differences (*n* = 3, ANOVA; *P* < 0.05). Typical deviation is represented by ± . < u.l. under detection limit

**Fig. 2:**
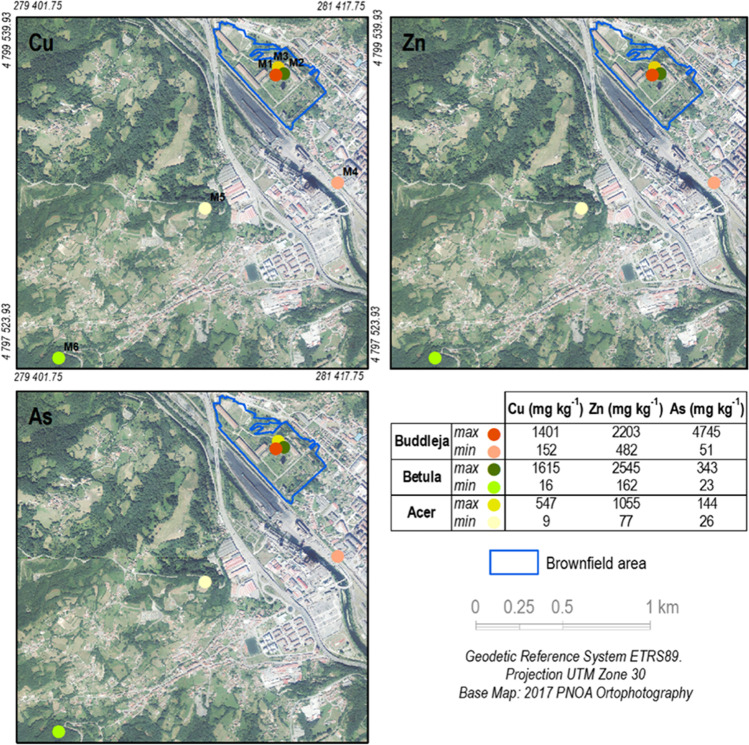
Graphical representation of pseudo-total concentrations of Cu, Zn, and As

As expected, soils M1, M2, and M3 showed significantly higher pseudo-total concentrations of Cu, Zn, and As than M4, M5, and M6 (Fig. 2, Table 3). These higher concentrations are attributed to the disposal, erosion, and blend of different types of waste, such as slag, coal waste, and pyrite ash, found throughout the brownfield with natural soil aggregates (Baragaño et al. 2020; Gallego et al. 2016).

**Table 3:** Pseudo-total concentrations of Cu, Zn, and As (mg.kg^−1^) in soils inside and outside the urban brownfield

Zone	Sample area	Plant species	Cu-pseudo-total	Zn-pseudo-total	As-pseudo-total
Inside	M1	*B. davidii*	1401.57 ± 33.82b	2202.65 ± 310.69b	4745.53 ± 95.87a
	M2	*B. celtiberica*	1615.17 ± 37.54a	2545.23 ± 147.82a	343.36 ± 9.59b
	M3	*A. pseudoplatanus*	546.86 ± 38.22c	1055.53 ± 48.95c	143.84 ± 7.63c
Outside	M4	*B. davidii*	151.71 ± 13.95d	481.87 ± 83.94d	51.35 ± 3.96d
	M5	*A. pseudoplatanus*	9.22 ± 0.70e	77.46 ± 5.53e	26.18 ± 1.93d
	M6	*B. celtiberica*	16.06 ± 3.33e	161.90 ± 35.95e	23.53 ± 0.22d

Different letters for distinct samples indicate significant differences (*n* = 3, ANOVA; *P* < 0.05). Typical deviation is represented by ± . < u.l. under the detection limit

Inside the brownfield, the highest concentrations of Cu and Zn were detected in soils encompassing the *B. celtiberica* sampling area, whereas As concentrations were the highest in the soils in which *B. davidii* was growing (Fig. 2). *B. celtiberica* can grow in soils with high concentrations of Cu and Zn (Fernández-Fuego et al. 2017a, 2017b). Other authors (Chaoyang et al. 2011) have also described *B. davidii* growth in soils with high concentrations of As, and, in general terms, it can grow in soils with considerable metal(loid) concentrations (Ge & Zhang 2014; Zhu et al. 2018). Coherently, outside the urban brownfield, the soils with the highest pseudototal concentrations of Cu, Zn, and As coincided with those in which *B. davidii* was growing (Fig. 2).

### Phytoavailable concentrations of Cu, Zn, and As

**Fig. 3:**
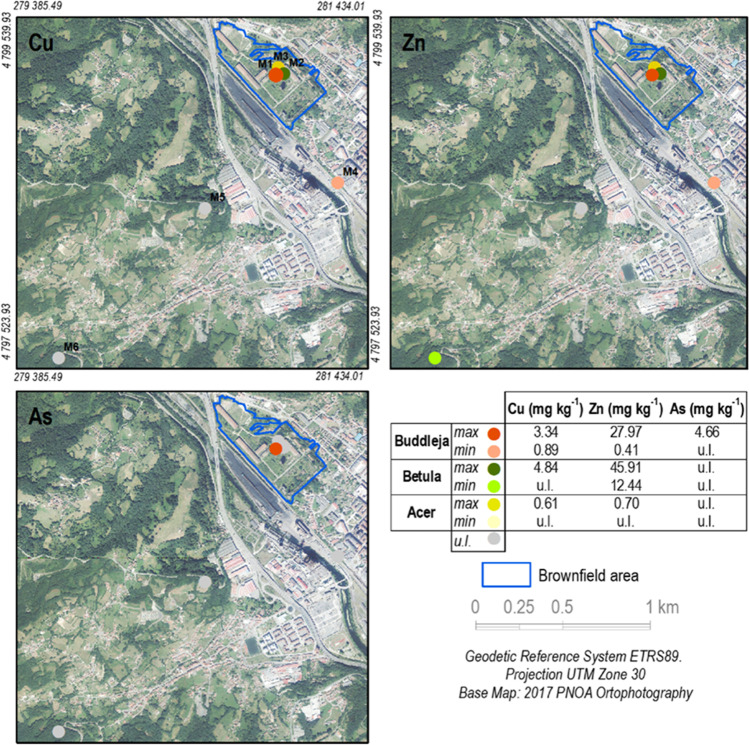
Graphical representation of phytoavailable concentrations of Cu, Zn, and As ((NH4)2SO4 extracted) in soil

**Fig. 4:**
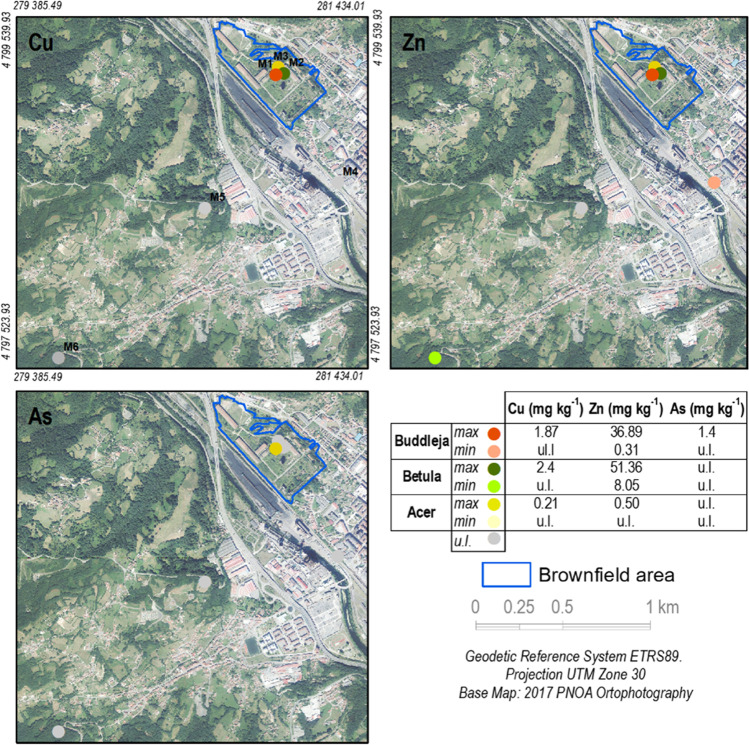
Graphical representation of the phytoavailable concentrations of Cu, Zn, and As (CaCl2 extracted) in soil

Phytoavailable concentrations of Cu, Zn, and As were higher inside the brownfield (M1, M2, M3 samples) irrespective of the extractants used (Figs. 3, 4). These high concentrations may be explained by the different types of residues, mainly pyrite ash, that were accumulated over time at this site (Gallego et al. 2016). In addition, the lower OM content and pH values within the brownfield soil (Table 2) may cause reduced sorption capacity compared to the natural soils outside the brownfield (Forján et al. 2016).

Regarding the proportions of extractable contaminants (Table 4), in general terms, the values were higher for Cu and Zn inside the brownfield, as was the case for As, although the latter showed very low values. The same, but to a greater extent, was observed for Pb. In this regard, notable pseudo-total Pb concentrations were previously reported (Gallego et al. 2016), but in the present study, we found that phytoavailable Pb was below the detection limit in all the samples examined, and thus, Pb data were not considered in this study. For more information, consult Table S1 (Supplementary material).

**Table 4:** Phytoavailable concentrations of Cu, Zn, and As (mg.kg^−1^) and their percentage vs pseudo-total concentrations in soils, inside and outside the urban brownfield

Element	Zone	Soil sample	Plant species	Extractable- (NH_4_)_2_SO_4_	Extractable-CaCl_2_	%extractable-(NH_4_)_2_SO_4_	%extractable-CaCl_2_
Cu	Inside	M1	*B. davidii*	3.34 ± 0.37b	1.87 ± 0.20b	0.23 ± 0.02bc	0.13 ± 0.01a
		M2	*B. celtiberica*	4.84 ± 1.80a	2.40 ± 0.64a	0.30 ± 0.11b	0.15 ± 0.04a
		M3	*A. pseudoplatanus*	0.61 ± 0.27c	0.21 ± 0.01c	0.11 ± 0.06c	0.04 ± 0.00b
	Outside	M4	*B. davidii*	0.89 ± 0.26c	u.l	0.58 ± 0.13a	u.l
		M5	*A. pseudoplatanus*	u.l	u.l	u.l	u.l
		M6	*B. celtiberica*	u.l	u.l	u.l	u.l
Zn	Inside	M1	*B. davidii*	27.97 ± 5.29b	36.89 ± 3.86b	1.27 ± 0.18b	1.69 ± 0.26b
		M2	*B. celtiberica*	45.91 ± 1.76a	51.36 ± 2.83a	1.81 ± 0.17b	2.02 ± 0.02b
		M3	*A. pseudoplatanus*	0.70 ± 0.13d	0.50 ± 0.02d	0.06 ± 0.01c	0.04 ± 0.00c
	Outside	M4	*B. davidii*	0.41 ± 0.07d	0.31 ± 0.09d	0.08 ± 0.02c	0.06 ± 0.02c
		M5	*A. pseudoplatanus*	u.l	u.l	u.l	u.l
		M6	*B. celtiberica*	12.44 ± 2.19c	8.05 ± 0.22c	7.93 ± 2.01a	5.15 ± 1.15a
As	Inside	M1	*B. davidii*	4.66 ± 0.51a	1.40 ± 0.13a	0.09 ± 0.01a	0.00
		M2	*B. celtiberica*	u.l	u.l	u.l	u.l
		M3	*A. pseudoplatanus*	u.l	u.l	u.l	u.l
	Outside	M4	*B. davidii*	u.l	u.l	u.l	u.l
		M5	*A. pseudoplatanus*	u.l	u.l	u.l	u.l
		M6	*B. celtiberica*	u.l	u.l	u.l	u.l

Different letters for different samples indicate significant differences (*n* = 3, ANOVA; *P* < 0.05). Typical deviation is represented by ± . < u.l. represent under the detection limit

Phytoavailable concentrations of metal(loid)s inside the brownfield could be attributed to the presence of soils mixed with the residues mentioned above, specifically pyrite ash, which is largely composed of oxides, hydroxides, and also sulfides of iron and other metal(loid)s, which were produced as by-products of the sulfide ore roasting process (Gallego et al. 2016; Mesa et al. 2017). As an exception, Zn in sample M6 (outside) showed a higher phytoavailable percentage, both with (NH_4_)_2_SO_4_ (7.93%) (Fig. 3) and CaCl_2_ extractants (5.15%) (Fig. 4), than that of any other sample inside the brownfield (< 2.10% for both extractants, Table 4). There could be various explanations for this observation, including the location of the different industries that have been operating in Langreo for more than a century and that left a heavy pollution footprint in the environmental compartments (Boente et al. 2022).

Specifically for the sampling stations of plants clusters, Zn presented the highest phytoavailable concentrations, both inside (0.11% with (NH_4_)_2_SO_4_ and 0.04% with CaCl_2_) and outside (7.93% with (NH_4_)_2_SO_4_ and 5.15% with CaCl_2_) the brownfield, coinciding with soils where *B. celtiberica* grew (Fig. 4, Table 4). Therefore, in the outside station, the percentage of extractable concentration versus pseudototal concentration was higher than inside the brownfield (Table 4) irrespective of the higher pseudototal concentration observed inside. This suggests that the mobility of Zn inside the brownfield is very low due to the pollution source (pyrite ash) as previously observed by Baragaño et al. (2020). A similar pattern was observed for phytoavailable concentrations of Cu inside the brownfield in the case of *B. davidii* with (NH_4_)_2_SO_4_ extraction (Fig. 3, Table 4). In contrast, the soils outside where *B. davidii* grew presented the highest concentrations of Cu, whereas phytoavailable As exceeded the detection limit values only in the inside area in which *B. davidii* grew (Fig. 4, Table 4).

### Metal(loid) concentrations in plants and plant/soil system

**Fig. 5:**
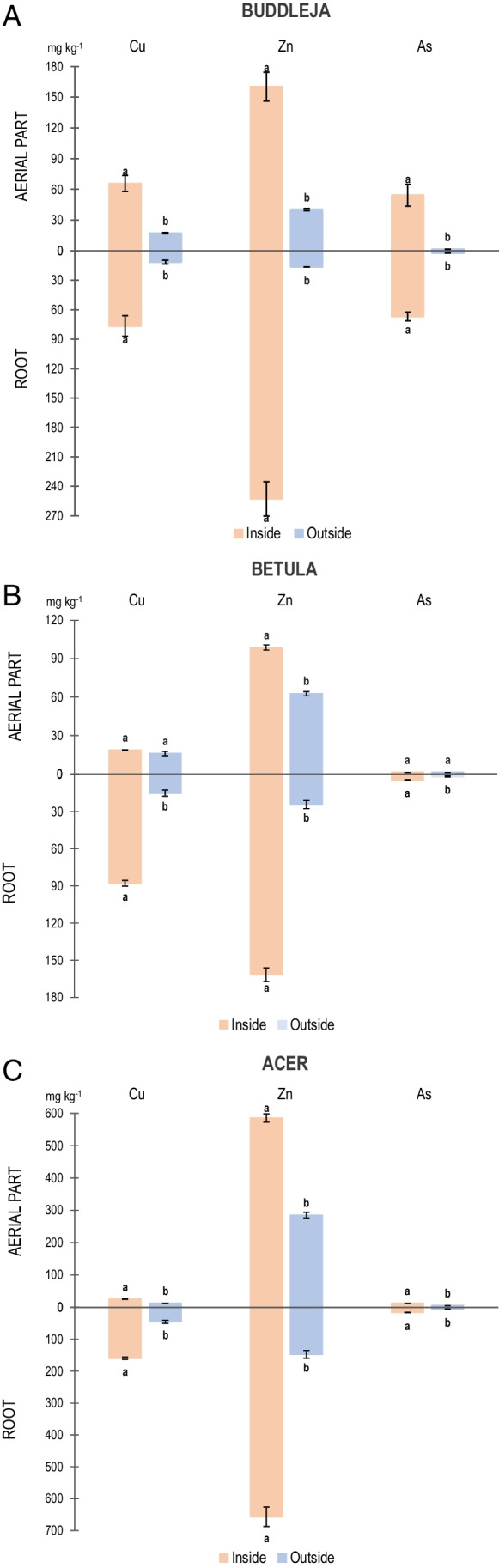
Metal(loid) concentrations in plants and plant/soil system. **A**. *B. davidii*, **B** *B. celtiberica*, and **C** *A. pseudoplatanus*. Locations inside the urban brownfield are indicated in orange and those outside in blue

In general, all the plant species presented higher concentrations of the metal(loids)s inside the brownfield than outside, both in the root and aerial part, with As presenting the lowest values and Zn the highest in all species (Fig. 5, Table S2 (Supplementary material)).

*B. davidii* sampled inside the urban brownfield had higher concentrations of Cu, Zn, and As (in roots and aerial part) compared to *B. davidii* sampled outside the urban brownfield (Fig. 5, Table S2). The area where *B. davidii* was collected revealed phytoavailable concentrations of Cu, Zn, and As that were significantly positively correlated (*p* < 0.01) with the contents of Cu, Zn, and As in the root and leaves of *B. davidii*.

Inside the urban brownfield, *B. celtiberica* presented higher contents of Cu, Zn, and As in both the root and aerial part than *B. celtiberica* outside the brownfield, except for As in the aerial part, although no significant differences were found (Fig. 5, Table S2). Phytoavailable Zn concentrations were significantly positively correlated with Zn contents in the root and aerial part of *B. celtiberica*. However, in the case of Cu, significant positive correlations were found only between phytoavailable Cu concentrations and root Cu content.

*A. pseudoplatanus* followed the same pattern as *B. davidii* and *B. celtiberica*. Inside the brownfield, *A. pseudoplatanus* had the highest concentrations of Cu, Zn, and As, both in the root and aerial part (Fig. 5, Table S2). These results are in concordance with those reported by authors such as Mleczek et al. (2017). Phytoavailable Zn concentrations were significantly positively correlated with Zn contents in the root and aerial part, and also with the BF values.

### Soil/vegetation indexes

The species studied inside the urban brownfield can be classified as accumulators or hyperaccumulators of metal(loid)s, presenting, in general, TC > 1 or proximal values according to Busuioc et al. (2011) (Table 5). In contrast, following the study by Baker and Brooks (1989), the species outside the brownfield present a high degree of meta(loid) translocation between soil and vegetation (Table 5).

**Table 5:** Cu, Zn, and As soil/vegetation factors (TF, TC, BFroot, BFaerial) in areas inside and outside the urban brownfield

Element	Zone	Soil sample	Plant species	TF	TC	BTFroot	BTFaerial
Cu	Inside	M1	*B. davidii*	0.84 ± 020	0.04 ± 0.00	23.88 ± 2.50	19.93 ± 4.51
		M2	*B. celtiberica*	0.21 ± 00	0.01 ± 0.00	20.06 ± 7.74	3.25 ± 0.46
		M3	*A. pseudoplatanus*	0.09 ± 0.00	0.02 ± 0.00	230.32 ± 55.52	34.65 ± 7.82
	Outside	M4	*B. davidii*	**1.55 ± 0.17**	0.11 ± 0.01	10.90 ± 1.93	17.25 ± 1.79
		M5	*A. pseudoplatanus*	0.18 ± 0.001	0.88 ± 0.03	u.l	u.l
		M6	*B. celtiberica*	**1.05 ± 0.07**	**1.01 ± 0.01**	u.l	u.l
Zn	Inside	M1	*B. davidii*	0.63 ± 0.09	0.07 ± 0.01	9.20 ± 1.36	5.91 ± 1.17
		M2	*B. celtiberica*	0.61 ± 0.03	0.03 ± 0.00	3.52 ± 0.07	2.15 ± 0.12
		M3	*A. pseudoplatanus*	0.89 ± 0.02	0.55 ± 0.02	1,063.08 ± 25.86	956.91 ± 36.26
	Outside	M4	*B. davidii*	**2.46 ± 0.10**	0.08 ± 0.01	36.13 ± 0.53	86.13 ± 0.36
		M5	*A. pseudoplatanus*	**1.93 ± 0.10**	**3.68 ± 0.25**	u.l	u.l
		M6	*B. celtiberica*	**2.58 ± 0.28**	0.39 ± 0.08	2.00 ± 0.41	5.15 ± 0.80
As	Inside	M1	*B. davidii*	0.82 ± 0.26	0.01 ± 0.00	14.43 ± 1.61	11.72 ± 3.26
		M2	*B. celtiberica*	u.l	u.l	u.l	u.l
		M3	*A. pseudoplatanus*	0.12 ± 0.04	u.l	u.l	u.l
	Outside	M4	*B. davidii*	u.l	u.l	u.l	u.l
		M5	*A. pseudoplatanus*	u.l	0.01 ± 0.00	u.l	u.l
		M6	*B. celtiberica*	u.l	u.l	u.l	u.l

Different letters indicate significant differences (*n* = 3, ANOVA; *P* < 0.05). Typical deviation is represented by ± . < u.l. under the detection limit. Bolded values indicate values higher than 1

*B. davidii* outside the urban brownfield had a TF > 1 for Cu and Zn (Table 5). In addition, the BF-aerial value for Cu and Zn was higher than the BFroot value, and the opposite was true inside the brownfield (Table 5). These values indicate that the behavior of *B. davidii* is distinct when inside and outside the urban brownfield. Outside the area, *B. davidii* has a high capacity to accumulate Cu and Zn in the aerial part, whereas inside the site, it accumulates these elements in the root, thereby suggesting phytostabilization capacity (Baker and Brooks 1989; Karami et al. 2011). The behavior of *B. davidii* that we observed in the soils with high concentrations of Cu and Zn is consistent with data reported by Zhu et al. (2018).

*B. celtiberica* inside the urban brownfield did not show TF or TC > 1 for any of the metal(oid)s analyzed. However, outside the area, it showed TF and TC > 1 for Cu and TF values higher than 1 for Zn. As in the case of *B. davidii*, the TF and TC values indicate two very different phytoremediation behaviors of *B. celtiberica*. Inside the brownfield, where phytoavailable concentrations are higher, *B. celtiberica* behaves as a phytostabilizing species, but outside, where concentrations are lower, it is a phytoextractive species. In fact, *B. celtiberica* is a fast-growing, deciduous, and pseudometallophilic tree. It has a high biomass and a well-developed root system. Although it has colonized the study area, it is usually found in restricted areas of the Iberian Peninsula (Shaw et al. 2014). Thus, the autoecology of this species suggests that it might be a suitable candidate to phytoremediate contaminated soils in Asturias (Mesa et al. 2017), like the urban brownfield examined herein. Authors such as Kříbek et al. (2020) concluded that *B. celtiberica* can grow on substrates with extremely high concentrations of trace elements and can therefore be used for phytoremediation purposes, especially on Zn-contaminated sites. In this regard, it should be noted that, in the urban brownfield studied here, Zn was the element with the highest pseudo-total and phytoavailable concentrations. Consequently, Zn was the metal that *B. celtiberica* accumulated the most (Table 5).

The TF and TC values exceeded 1 only in *A. pseudoplatanus* outside the brownfield, although it should be noted that these values did not exceed 1 for Cu in this species either inside or outside the brownfield (Table 5). For Zn, the BF values inside the site and the TF and TC values outside indicate the high capacity of *A. pseudoplatanus* to phytoremediate Zn-contaminated soils. In addition, *A. pseudoplatanus* can enhance the reduction of metal(loid) concentrations in the soil as it has good litter quality, which promotes rapid decomposition, lower production of acids, and the formation of stable humus (Reich et al. 2005). Another positive feature of *A. pseudoplatanus* is that lower amounts of Zn are found in the litter it produces compared to other phytoremediation species (Mertens et al. 2007).

## Conclusions

Spontaneously growing species showed a high capacity for adaptation to the environmental conditions. The phytoavailable concentrations of metal(loid)s showed that concentrations were higher inside the brownfield than outside. However, the TF and TC indicated that the species studied outside the brownfield, on average, had phytoextractive capacity and that those inside the brownfield had phytostabilization capacity. Thus, on the basis of the results obtained from the indexes related to phytoremediation, *A. pseudoplatanus*, *B. celtiberica*, and *B. davidii* follow different phytoremediation strategies depending on the degree of contamination of the soil. Therefore, for real-scale treatments, the three species studied herein emerge as candidates for phytostabilization actions in areas with high levels of contaminants, whereas their phytoextraction capacity is suitable only for soils with low levels of pollution.

## Supplementary information

Below is the link to the electronic supplementary material.

Supplementary file1 (DOCX 16 KB)

## Data Availability

Data is available on reasonable request from the corresponding author.

## References

[CR1] Ali H, Khan E, Sajad MA (2013). Phytoremediation of heavy metals—concepts and applications. Chemosphere.

[CR2] Asensio V, Abreu-Junior CH, da Silva FC, Chitolina JC (2018). Evaluation of chemical extractants to assess metals phytoavailability in Brazilian municipal solid waste composts. Environ Pollut.

[CR3] Baker AJM, Brooks RR (1989) Terrestrial higher plants which hyperaccumulate metal elements. A review of their distribution, ecology and phytochemistry – ScienceOpen. Biorecovery. 1:81–126. https://www.scienceopen.com/document?vid=ee525bc2-564c-4191-93a3-ab0265138a85

[CR4] Baragaño D, Forján R, Fernández B, Ayala J, Afif E, Gallego JLR (2020). Application of biochar, compost and ZVI nanoparticles for the remediation of As, Cu, Pb and Zn polluted soil. Environ Sci Pollut Res.

[CR5] Baragaño D, Gallego JL, Forján R (2021). Short-term experiment for the in situ stabilization of a polluted soil using mining and biomass waste. J Environ Manage..

[CR6] Boente C, Albuquerque MTD, Gallego JR, Pawlowsky-Glahn V, Egozcue JJ (2022). Compositional baseline assessments to address soil pollution: an application in Langreo. Spain. Sci Total Environ.

[CR7] BOPA, Boletín Oficial del Principado de Asturias (2014) Generic reference levels for heavy metals in soils from principality of Asturias, Spain. https://sede.asturias.es/bopa/ 2014/04/21/2014–06617.pdf, Accessed date: 1 August 2019 (91, April 21)

[CR8] Busuioc G, Cristina Elekes C, Stihi C, Iordache S, Constantin CS (2011). The bioaccumulation and translocation of Fe, Zn, and Cu in species of mushrooms from Russula genus. Environ Sci Pollut Res.

[CR9] Chaoyang W, Deng Qiujing Wu, Ziyou FF, Libin Xu, Wei C, Deng Q, Wu F, Fu Z, Xu L (2011). Arsenic, antimony, and bismuth uptake and accumulation by plants in an old antimony mine. China Biol Trace Elem Res.

[CR10] Fernández-Fuego D, Bertrand A, González A (2017). Metal accumulation and detoxification mechanisms in mycorrhizal Betula pubescens. Environ Pollut.

[CR11] Fernández-Fuego D, Keunen E, Cuypers A, Bertrand A, González A (2017). Mycorrhization protects Betula pubescens Ehr. from metal-induced oxidative stress increasing its tolerance to grow in an industrial polluted soil. J Hazard Mater.

[CR12] Forján R, Asensio V, Rodríguez-Vila A, Covelo EF (2016). Contribution of waste and biochar amendment to the sorption of metals in a copper mine tailing. CATENA.

[CR13] Forján R, Rodríguez-Vila A, Covelo EF (2018). Using compost and technosol combined with biochar and Brassica juncea L. to decrease the bioavailable metal concentration in soil from a copper mine settling pond. Environ Sci Pollut Res.

[CR14] Forján R, Rodríguez-Vila A, Pedrol N, Covelo EF (2017). Application of compost and biochar with Brassica juncea L. to reduce phytoavailable concentrations in a settling pond mine soil. Waste Biomass Valorization.

[CR15] Fresno T, Moreno-Jiménez E, Peñalosa JM (2016). Assessing the combination of iron sulfate and organic materials as amendment for an arsenic and copper contaminated soil. Chem Ecotoxicological Approach Chemosphere.

[CR16] Gallego JR, Rodríguez-Valdés E, Esquinas N, Fernández-Braña A, Afif E (2016). Insights into a 20-ha multi-contaminated brownfield megasite: an environmental forensics approach. Sci Total Environ.

[CR17] Ge J, Zhang J (2014). Heavy metal contamination and accumulation in soil and plant species from the Xinqiao copper deposit, Anhui Province. China Anal Lett.

[CR18] Gil-Díaz M, Diez-Pascual S, González A, Alonso J, Rodríguez-Valdés E, Gallego JR, Lobo MC (2016). A nanoremediation strategy for the recovery of an As-polluted soil. Chemosphere.

[CR19] GuidiNissim W, Labrecque M (2021). Reclamation of urban brownfields through phytoremediation: implications for building sustainable and resilient towns. Urban For Urban Green..

[CR20] Hou D, Song Y, Zhang J, Hou M, O’Connor D, Harclerode M (2018). Climate change mitigation potential of contaminated land redevelopment: a city-level assessment method. J Clean Prod.

[CR21] Houba VJG, Temminghoff EJM, Gaikhorst GA, van Vark W (2008). Soil analysis procedures using 0.01 M calcium chloride as extraction reagent. Commun Soil Sci Plant Anal..

[CR22] Karami N, Clemente R, Moreno-Jiménez E, Lepp NW, Beesley L (2011). Efficiency of green waste compost and biochar soil amendments for reducing lead and copper mobility and uptake to ryegrass. J Hazard Mater.

[CR23] Kidd P, Barceló J, Bernal MP, Navari-Izzo F, Poschenrieder C, Shilev S, Clemente R, Monterroso C (2009). Trace element behaviour at the root–soil interface: implications in phytoremediation. Environ Exp Bot.

[CR24] Kříbek B, Míková J, Knésl I, Mihaljevič M, Sýkorová I (2020). Uptake of trace elements and isotope fractionation of Cu and Zn by birch (Betula pendula) growing on mineralized coal waste pile. Appl Geochemistry..

[CR25] Lebourg A, Sterckeman T, Ciesielski H, Proix N, Gomez A (2010). Estimation of soil trace metal bioavailability using unbuffered salt solutions: degree of saturation of polluted soil extracts. Environ Technol.

[CR26] Massenet A, Bonet A, Laur J, Labrecque M (2021). Co-planting Brassica napus and Salix nigra as a phytomanagement alternative for copper contaminated soil. Chemosphere..

[CR27] Matanzas N, Afif E, Díaz TE, Gallego JR (2021) Phytoremediation potential of native herbaceous plant species growing on a paradigmatic brownfield site. Water Air Soil Pollut 232(7):290. 10.1007/s11270-021-05234-9

[CR28] McGrath SP, Zhao F (2003). Phytoextraction of metals and metalloids from contaminated soils. Curr Opin Biotechnol.

[CR29] Menzies NW, Donn MJ, Kopittke PM (2007). Evaluation of extractants for estimation of the phytoavailable trace metals in soils. Environ Pollut.

[CR30] Mertens J, Van Nevel L, De Schrijver A, Piesschaert F, Oosterbaan A, Tack FMG, Verheyen K (2007). Tree species effect on the redistribution of soil metals. Environ Pollut.

[CR31] Mesa V, Navazas A, González-Gil R, González A, Weyens N, Lauga B, Luis J, Gallego R, Sánchez J, Peláez AI (2017). Use of endophytic and rhizosphere bacteria to improve phytoremediation of arsenic-contaminated industrial soils by autochthonous. Betula Celtiberica.

[CR32] Midhat L, Ouazzani N, Esshaimi M, Ouhammou A, Mandi L (2016). Assessment of heavy metals accumulation by spontaneous vegetation: screening for new accumulator plant species grown in Kettara mine-Marrakech. Southern Morocco.

[CR33] Mleczek M, Goliński P, Krzesłowska M, Gąsecka M, Magdziak Z, Rutkowski P, Budzyńska S, Waliszewska B, Kozubik T, Karolewski Z, Niedzielski P (2017). Phytoextraction of potentially toxic elements by six tree species growing on hazardous mining sludge. Env Sci Pollut Res.

[CR34] Mukhopadhyay S, Rana V, Kumar A, Maiti SK (2017). Biodiversity variability and metal accumulation strategies in plants spontaneously inhibiting fly ash lagoon. India Environ Sci Pollut Res.

[CR35] O’Connor D, Zheng X, Hou D, Shen Z, Li G, Miao G, O’Connell S, Guo M (2019). Phytoremediation: climate change resilience and sustainability assessment at a coastal brownfield redevelopment. Environ Int.

[CR36] Peijnenburg WJG, Jager T (2003). Monitoring approaches to assess bioaccessibility and bioavailability of metals: matrix issues. Ecotox Environ Safe.

[CR37] Pilon-Smits E (2005). Phytoremediation. Ann Rev. Plant Biol.

[CR38] Reich PB, Oleksyn J, Modrzynski J, Mrozinski P, Hobbie SE, Eissenstat DM, Chorover J, Chadwick OA, Hale CM, Tjoelker MG (2005). Linking litter calcium, earthworms and soil properties: a common garden test with 14 tree species. Ecol Lett.

[CR39] Rey E, Laprise M, Lufkin S (2022) Urban brownfield regeneration projects: complexities and issues. In: Neighbourhoods in Transition. The Urban Book Series. Springer, Cham. 10.1007/978-3-030-82208-8_4

[CR40] Rodríguez-Vila A, Covelo EF, Forján R, Asensio V (2014). Phytoremediating a copper mine soil with Brassica juncea L., compost and biochar. Environ Sci Pollut Res..

[CR41] Rodríguez-Vila A, Covelo EF, Forján R, Asensio V (2015). Recovering a copper mine soil using organic amendments and phytomanagement with Brassica juncea L. J Environ Manage.

[CR42] Rodríguez-Vila A, Forján R, Guedes RS, Covelo EF (2016) Changes on the phytoavailability of nutrients in a mine soil reclaimed with compost and biochar. Water Air Soil Pollut 227:453. 10.1007/s11270-016-3155-x

[CR43] Schat H, Llugany M, Bernhard R (2020) Metal-specific patterns of tolerance, uptake, and transport of heavy metals in hyperaccumulating and nonhyperaccumulating metallophytes. In: Terry N, Bañuelos G (eds) Phytoremediation of contaminated soil and water Phytoremediation. Taylor and Francis, UK, pp 178–195 10.1201/9780367803148-9

[CR44] Shaw K, Stritch L, Rivers M, Roy S, Wilson B, Govaerts R (2014) The red list of Betulaceae (Book). Botanic Gardens Conservation International, Richmond, UK. https://globaltrees.org/wp-content/uploads/2014/11/Betulace8-FINAL.pdf

[CR45] Wcislo E, Bronder J, Bubak A, Rodríguez-Valdés E, Gallego JR (2016). Human health risk assessment in restoring safe and productive use of abandoned contaminated sites. Environ Int.

[CR46] Zhu G, Xiao H, Guo Q, Song B, Zheng G, Zhang Z, Zhao J, Okoli CP (2018). Heavy metal contents and enrichment characteristics of dominant plants in wasteland of the downstream of a lead-zinc mining area in Guangxi. Southwest China Ecotoxicol Environ Saf.

