# Supplementary material for: Phytoremediation potential depends on the degree of soil pollution: a case study in an urban brownfield
Source: Environ Sci Pollut Res Int. 2023 Apr 28;30(25):67708–19. doi: 10.1007/s11356-023-26968-5 (PMC10203031; doi:10.1007/s11356-023-26968-5)
Supplement: Supplementary file 1 — Supplementary file1 (DOCX 16 KB) [file 11356_2023_26968_MOESM1_ESM.docx]

**Supplementary material**

Table S1. Concentrations of Cu, Zn and As (mg.kg^-1^) in soils inside and outside the urban brownfield in roots and aerial part of the vegetation.

| **Element** | **Zone** | **Soil sample** | **Plant species** | **Concentration in root** | **Concentration in aerial part** |
| --- | --- | --- | --- | --- | --- |
| Cu | Inside | M1 | *B. davidii* | 76.65±10.57a | 65.61±7.74a |
|  |  | M2 | *B. celtiberica* | 87.94±2.28a | 18.65±0.39a |
|  |  | M3 | *A. pseudoplatanus* | 159.82±3.64a | 15.11±0.73a |
|  | Outside | M4 | *B. davidii* | 11.28±1.83b | 17.36±0.06b |
|  |  | M5 | *A. pseudoplatanus* | 45.08±5.16b | 8.11±0.35b |
|  |  | M6 | *B. celtiberica* | 15.21±2.58b | 15.99±1.62a |
| Zn | Inside | M1 | *B. davidii* | 252.78±17.47a | 160.24±24.03a |
|  |  | M2 | *B. celtiberica* | 161.73±5.34a | 98.66±1.94a |
|  |  | M3 | *A. pseudoplatanus* | 657.08±30.61a | 585.37±12.83a |
|  | Outside | M4 | *B. davidii* | 16.34±0.22b | 40.28±1.17b |
|  |  | M5 | *A. pseudoplatanus* | 147.62±12.02b | 284.68±8.54b |
|  |  | M6 | *B. celtiberica* | 24.47±3.18b | 62.68±1.70b |
| As | Inside | M1 | *B. davidii* | 66.92±4.52a | 54.28±13.66a |
|  |  | M2 | *B. celtiberica* | 3.56±0.28a | 0.13±0.01a |
|  |  | M3 | *A. pseudoplatanus* | 3.72±0.62a | 0.45±0.07a |
|  | Outside | M4 | *B. davidii* | 0.24±0.01b | 0.48±0.12b |
|  |  | M5 | *A. pseudoplatanus* | 1.56±0.38b | 0.29±0.02b |
|  |  | M6 | *B. celtiberica* | 0.82±0.44b | 0.15±0.03a |

*Different letters for different samples indicate significant differences (n=3, ANOVA; P<0.05). Typical deviation is represented by ±. < u.l. represent under detection limit.*
